# Cancer in Kenya: types and infection-attributable. Data from the adult population of two National referral hospitals (2008-2012)

**DOI:** 10.12688/aasopenres.12910.5

**Published:** 2019-11-14

**Authors:** Lucy Wanjiku Macharia, Marianne Wanjiru Mureithi, Omu Anzala

**Affiliations:** 1 Department of medical Microbiology, Faculty of Medicine, University of Nairobi, Nairobi, Kenya; 2 KAVI-Institute of Clinical Research (KAVI-ICR), College of Health Sciences, University of Nairobi, Nairobi, Kenya

**Keywords:** Cancer burden, infectious agents, sub-Saharan Africa, Kenya

## Abstract

**Background:** Cancer in Africa is an emerging health problem. In Kenya it ranks third as a cause of death after infectious and cardiovascular diseases. Nearly 31% of the total cancer burden in sub-Saharan Africa is attributable to infectious agents. Information on cancer burden is scanty in Kenya and this study aimed to provide comprehensive hospital based data to inform policies.

**Method: **A cross-sectional retrospective survey was conducted at Kenyatta National Hospital (KNH) and Moi Teaching and Referral Hospital (MTRH) from January 2008 to December 2012.  Data was obtained from the patients files and the study was approved by the KNH/University of Nairobi and MTRH Ethics and Research Committees.

**Results**: In KNH, the top five cancers were: cervical (62, 12.4%), breast (59, 11.8%), colorectal (31, 6.2%), chronic leukemia (27, 5.4%) and stomach cancer (26, 5.2%). Some 154 (30.8%) of these cancers were associated with infectious agents, while an estimated 138 (27.6%) were attributable to infections. Cancers of the cervix (62, 12.4%), stomach (26, 5.2%) and nasopharynx (17, 3.4%) were the commonest infection-associated cancers. In MTRH, the five common types of cancers were Kaposi’s sarcoma (93, 18.6%), breast (77, 15.4%), cervical (41, 8.2%), non-Hodgkin’s lymphoma (37, 7.4%) and colorectal, chronic leukemia and esophageal cancer all with 27 (5.4%). Some 241 (48.2%) of these cancers were associated with infectious agents, while an estimated 222 (44.4%) were attributable to infections. Kaposi’s sarcoma (93, 18.6%), cancer of the cervix (41, 8.2%) and non-Hodgkin’s lymphoma (37, 7.4%) were the commonest infection-associated cancers.

**Conclusion: **Our results suggest that ****30.8% and 48.2% of the total cancer cases sampled in KNH and MTRH respectively were associated with infectious agents, while 27.6% and 44.4% were attributable to infections in the two hospitals respectively. Reducing the burden of infection-attributable cancers can translate to a reduction of the overall cancer burden.

## Background

Cancer in Africa is an emerging health problem where about 847,000 new cancer cases and 591,000 deaths occurred in 2012, with about three quarters of these occurring in the sub-Saharan region ^1^. In Kenya, cancer ranks third as a cause of death, after infectious and cardiovascular diseases, and in 2012 there was an estimated 37,000 new cancer cases, and 28,500 cancer deaths reported ^2^. Infectious agents are an important cause of cancer, particularly in less developed countries. According to the International Agency for Research on Cancer (IARC), 11 infectious agents have been classified and established as carcinogenic agents in humans namely: *Helicobacter pylori*, hepatitis B virus (HBV), hepatitis C virus (HCV), human immunodeficiency virus type 1 (HIV-1), human papillomavirus (HPV), Epstein-Barr virus (EBV), human herpes virus type 8 (HHV-8; also known as Kaposi’s sarcoma herpes virus), human T-cell lymphotropic virus type 1 (HTLV-1), *Opisthorchis viverrini*, *Clonorchis sinensis*, and *Schistosoma haematobium* ^3^.

Nearly 31% of the total cancer burden in sub-Saharan Africa is attributable to infections ^4^. Specifically, *H. pylori*, HPV, HBV, and HCV are the leading infectious agents contributing to the global cancer burden. When summed together they account for 92% of all infection-attributable cancers worldwide with 35.4%, 29.5%, 19.2%, and 7.8% respectively ^4^. The rise of the HIV epidemic concentrated in low and middle-income countries has resulted to an increase in HIV-associated malignancies ^5^. In Kenya, a HIV prevalence of 5.6% (95% CI: 4.9 to 6.3), and HIV incidence of 0.5% (95% CI: 0.2 to 0.9), corresponding to an annual HIV transmission rate of 8.9 per 100 HIV-infected persons has been reported ^6^. This population is at greater risk of acquiring HIV associated cancers such as Kaposi’s sarcoma (KS), non-Hodgkin lymphoma (NHL) and invasive cancer of the cervix (ICC) ^7^. According to a recent study, KS was the second largest contributor to the cancer burden in sub-Saharan Africa ^4^, while NHL is the second most common malignant disorder associated with HIV infection worldwide ^7^. Previous studies done in Africa have been discussed in details in the methodology section.

Information on the burden of cancer and especially the burden attributable to infections is sparse in Kenya. In this study, we highlight the results from the adult population of two National referral hospitals in Kenya for five-year period between January 2008 to December 2012.

## Methods

This was a retrospective cross-sectional study conducted at Kenyatta National Hospital (KNH) and Moi Teaching and Referral Hospital (MTRH) in Kenya. Initially the study targeted four teaching and referral hospitals located in the former Nairobi, Rift Valley, Coast and Nyanza Provinces, but the authorization to access medical records was only granted by the above mentioned hospitals. Kenya was divided into eight provinces ( see map from the Kenya bureau of statistics ^8^) before the new constitution of Kenya that came into force in 2013. KNH is located in the former Nairobi Province which is the capital and the largest city of Kenya and according to the last official census taken in 2009 it had a population of 3,138,369 whose number has since grown to approximately 3.5 million people. KNH has a capacity of 2,000 beds and attends to an annual average of 70,000 inpatients and 600,000 outpatients. The Hospital has 50 wards and 24 operating theatres as well as 24 consultant clinics with over 6000 staff members. As a referral hospital, KNH offers specialized quality health care to patients from all over the Nation, East and Central Africa Region. MTRH is located in the former Rift Valley Province (Kenya’s largest Province) with a population of 10,006,805 ^8^. MTRH has a bed capacity of about 991 patients, an average number of 1200 patients at any time and about 1500 out patients per day. The Hospital serves residents of Western Kenya Region (representing at least 22 Counties), parts of Eastern Uganda and Southern Sudan. AMPATH-Oncology centre is located at MTRH which evolved from a HIV program to a pediatric cancer program and later to cancer care program. It is integrated in the MTRH department of Hematology and Oncology ^9^. At AMPATH oncology centre more than 1000 cancer patients are treated each month with about 45 oncology specialists. According to the Kenyan network of cancer organizations KNH and MTRH are amongst the oldest and largest public referral hospitals with cancer treatment services. Both hospitals offer screening, diagnosis and treatment services. There are few radiation machines and according to Strother *et al.*, 2013, there were two cobalt-60 radiation oncology machines, both housed in KNH ^9^. The two national hospitals are also the largest source of data for the two main cancer registries in Kenya. KNH provides data to the Nairobi cancer registry located in Nairobi while MTRH provides data to the Eldoret cancer registry located in Rift Valley. Recently, with an aim to decongest the national hospitals more (private, mission and public) health facilities have been equipped with the cancer services. However, because of the affordability of the services many patients opt for the public health facilities.

### Data source

Data for this study was obtained from hospital records of patients as this was the most convenient data source for all the information targeted.

**Inclusion criteria**
• Hospital records of patients diagnosed with cancer during the period January 2008 to December 2012.
• Records of patients above the age of 18 at the time of diagnosis and with a confirmed diagnosis either by histology, radiology or haematology.
**Exclusion criteria**
• Hospital records with incomplete data or not meeting the above criteria.

### Sample size calculation

The sample size (n) was calculated according to the guidelines outlined for calculating sample sizes for cross-sectional studies (qualitative variable) as explained by 10– 12 and as shown below;

$n=\frac{z^{2}\;\hat{p}(1-\hat{p})}{d^{2}}$

      Where:

**p** = Prevalence of condition or health state or the expected prevalence or proportion or estimated proportion of a disease

**d** = degree of precision of the estimate or the absolute error

**z** = Z statistic for a level of confidence or is the normal distribution critical value for a probability of α/2 in each tail. For a 95% CI, z=1.96. A 95% level of confidence and a ±5% (0.05) degree of precision were considered.

The prevalence of cancer in Kenya was not known at the time of the study and therefore a prevalence of 50% (0.5) was used in calculating the sample size. Elsewhere, it has been highlighted that when d=0.05 and a z=1.96, using a p of 0.5 (50%) yields the highest estimates for n (sample size) ^13^.

A sample size of 384 was estimated as the minimal necessary to achieve the required power of the study. As the data from this study was collected at a single point in time, a 30% (116) non-response allowance was factored in resulting to a final sample size of 500. This allowance would help us in case one of the 384 files would be found incomplete after the completion of the data collection period. In KNH an estimated 17,584 (inpatient and outpatient) cancer files were reported while in MTRH 4304 (inpatient) cancer cases were reported during the five year period. Due to cost and time constraints, it was only feasible to study 500 files as calculated from the sample size. To obtain the final number of 500 files from the totals in each hospital, a proportional stratified sampling method was used as described by 11. (see Table 1)

**Table 1.** Sample calculations for Kenyatta National Hospital (KNH) and Moi Teaching and Referral Hospital (MTRH).

	KNH			MTRH		
Year	Estimated Files (N)	Sampling fraction	Final sample size (Proportion)	Estimated Files (N)	Sampling fraction	Final sample size (Proportion)
2008	3168	18%	90	947	22%	110
2009	2834	16%	80	1325	31%	154
2010	3048	17%	85	489	11%	57
2011	4161	24%	120	732	17%	85
2012	4373	25%	125	811	19%	94
**Total**	**17,584**	**100%**	**500**	**4,304**	**100%**	**500**

To randomly select the calculated proportions of files for each year obtained in the previous step, a systematic random sampling method was used as described by 11. At KNH, the files were all available at the health information department and in the databases where systematic random sampling was an automated process. At the time of data collection, MTRH was in the process of updating the database and only 2012 files were available at the health information department. The files for 2008 to 2011 were obtained from the oncology centre at the Academic Model for the Prevention and Treatment of HIV/AIDS (AMPATH), and convenient sampling was used to achieve the required number of files. The reason for choosing convenient sampling was because we could not establish the total sampling frame to do the systematic randomization. However, an indirect systematic randomization was applied to select the files needed from the total files accessed per year and not the sampling frame. By the end of the data collection period, all the targeted 500 files were available from both the hospitals. In case a selected file was found incomplete during the data collection period, it was replaced by the next file following it after randomization.

### Data collection

A pre-designed questionnaire was used to abstract the information ( Supplementary File 1). The information abstracted from the files included patients age, sex, origin, type of cancer, method of cancer diagnosis, year of diagnosis and whether the patient was referred from another hospital.

### Ethical consideration

The study was approved by the Kenyatta National Hospital/University of Nairobi (KNH/UoN-ERC) and the Moi Teaching and Referral Hospital (IREC/MTRH) Ethics and Research Committees with approval numbers P24/01/2013 and FAN:IREC 1027 respectively. Endorsement for the study was obtained from the Director of Medical Services while the permission to access data from the hospital databases and patients files was obtained from the director of the Health Information Department. Although, the study aimed at conducting the research at four referral hospitals in Kenya, only two hospitals granted permission to access the patient files while the other two refused and the reasons for refusal were unknown. The study was a minimal risk study and patient consent was not sought since there was no direct patient involvement but a retrospective review of patients’ files. However, the patient identifying information was not included in the data collection forms.

### Data handling and analysis

Data was entered into statistical package for social sciences programme (IBM-SPSS) version 23 that was labeled using the exact fields as the questionnaires and the excel files. Quality control checks were performed to prevent double entry and to ensure accurate entry of the data. The proportions of cancer cases were analyzed with reference to the study site, sex and the age group. GraphPad Prism 6 (GraphPad Software Inc., San Diego, CA, USA) was used to draw the figure images. The cancers were listed according to the third edition of the International Classification of Diseases for Oncology (ICD-O). For cancers like BL (9687/3), HL (9650/3), NHL (9596/3), adult T-cell leukemia (9827/3), KS (9140/3), acute leukemia (9835/3 & 9861/3), chronic leukemia (9863/3 & 9823/3) and multiple myeloma (9732/3) where ICD-0-3 topographical code was not clear, we converted the IDC-0-3 morphological code to ICD10 topographical code for clarity.

Although many studies have shown a variety of cancers associated with infectious agents, the attributable fraction (AF) standard formula was applied to a group of cancers classified as carcinogenic in the IARC monograph 100b ^3^ namely; cervix (C53), liver (C22.0), stomach (C16), Kaposi’s sarcoma (C46), non-Hodgkin’s lymphoma (C82-85, C96), Hodgkin’s lymphoma (C81), nasopharynx (C11), oropharynx (C10), bladder (C67), vulva (C51), vagina (C52), penis (C60), anus (C21) and bile duct (C22.1). Calculation of the attributable fraction (AF) relies upon the standard formula for population attributable risk as shown by 14, 15:

AF= *p*( *r*-1) / [ *p*( *r*-1)+1]

Where:

p=prevalence of exposure to the infectious agents to the population

r=relative risk of exposure

However, the use of this formula requires prior knowledge on *p* and *r* as defined above and since this information is limited in Kenya, we used AF derived from other studies done elsewhere either in developing countries, sub-Saharan Africa, or world estimates that used data from developing countries. The formula results in a proportion that is applied to the total number of cases in the target population to obtain the number of cases that can theoretically be attributed to the factor in that population ^14^.

In determining AF estimates for cervical cancer, we identified other African studies that reported the prevalence of HPV in cervical cancer to be between 76% to 91% ^16– 18^. However, an AF of 100% was used for this study since the causality of HPV in cervical cancer is generally known ^4, 19, 20^. For vulvar cancer, studies from Botswana reported a HPV prevalence of 56.8 to 100% ^21, 22^, but an AF of 40% derived from developing countries estimates was used ^14^. We did not find any clear study showing the prevalence of HPV in vaginal cancers in sub-Saharan Africa, but an AF of 78% derived from world estimates was used ^4, 20^. Studies from Central African Republic and Botswana identified a prevalence of HPV in anal cancer to be 69.1% ^21, 23^, but a world estimate AF of 88% was used ^4, 20^. The prevalence of HPV in penile cancer has been shown to be 41.9% to 68% from Kenya and Botswana studies ^21, 24^ but a world estimate AF of 50% was used ^4, 20^. A study from South Africa showed the prevalence of HPV in oropharyngeal cancer to be 5.6% among men ( *N*=125) ^25^, while another from Ghana found a HPV prevalence of 13.8% (4 of 29) from the oral cavity, and 18.2% (2 of 11) from the pharynx ^26^, but an AF of 30.8% derived from world estimate was used ^4, 20^ ( Table 2).

**Table 2.** Previous prevalence studies on infectious agents and cancers.

Infectious agent	Cancer Site	ICD-0 code	Studies done in sub Saharan Africa	Prevalence of the infectious agent (%)	AF estimates used for this study (%)
HPV	Cervix uteri	C53	South Africa, Gabon ^16– 18^	76–91	100 ^4, 19, 20^
	Vulva	C51	Botswana ^21, 22^	57–100	40 ^14^
	Vagina	C52	-	-	78 ^4, 20^
	Anus	C21	Central African Republic, Botswana ^21, 23^	69	88 ^4, 20^
	Oropharynx	C10	South Africa, Ghana ^25, 26^	6–18	31 ^4, 20^
	Penis	C60	Kenya, Botswana ^21, 24^	42–68	50 ^4, 20^
HHV8/HIV	KS	**C46**	Kenya ^27^	50–64	100 ^4, 7, 20^
EBV	HL	**C81**	Kenya, Zambia, Malawi ^28– 30^	41–100	74 ^4^
	Nasopharynx	C11	-	-	96 ^4^
	BL	C83.7	Africa ^31^	-	-
EBV/HIV	NHL	**C82-C85,** **C96**	Zambia ^28^	55	100 ^14, 15^
HBV/HCV	Liver	C22.0	Gambia ^32^	70	71 ^33^
*H. pylori*	Stomach	C16	Nigeria, Uganda ^34, 35^	82–86	89 ^4^
*Schistosoma*	Bladder	C67	South Africa ^36^	10–85	41 ^4^
*O. viverrini* *C. sinensis*	Bile duct	C22.1	-		-
*HTLV-1*	ATLL	C91.5	-	-	-

*Helicobacter pylori* (H. pylori), hepatitis B virus (HBV), hepatitis C virus (HCV), human immunodeficiency virus type 1 (HIV-1), human papillomavirus (HPV), Epstein-Barr virus (EBV), human herpes virus type 8 (HHV-8), human T-cell lymphotropic virus type 1 (HTLV-1), Adult T-cell Leukaemia/lymphoma (ATLL), *Opisthorchis viverrini*, *Clonorchis sinensis*, *Schistosoma haematobium*, non-Hodgkin’s lymphoma (NHL), Kaposi’s sarcoma (KS), Hodgkin’s lymphoma (HL) Burkitt’s lymphoma (BL), AF=attributable fraction, - = not available or not obtainable

In determining AF estimates for KS, a study from Western Kenya found a Kaposi´s sarcoma-associated herpes virus (KSHV) positivity of 50.1 to 63.5% using 228 surgical cases ^27^ but a world estimate AF of 100% was used since HHV8 is recognized as a necessary cause of KS in HIV infections ^4, 7^. For EBV in NHL, A study from Lusaka, Zambia, detected EBV in 54.5% of the cases ^28^. We used an estimated AF of 100% based on meta-analysis studies from developing countries ^14, 15^. Elsewhere in Malawi, EBV in HL was present in 18 of 24 (75%) tumor specimens, including 14 of 20 (70%) HIV- and 4 of 4 (100%) HIV+ ^29^. Similarly a study from Kenya found an EBV in HL positivity of 100% present in pediatric cases ^30^ while Zambia, detected EBV in HL of 40.9% ^28^. However, an AF of 74% derived from African estimates was used ^4^. No studies were encountered showing the prevalence of EBV in nasopharyngeal cancer in sub-Saharan Africa, therefore an AF of 95.5% derived from world estimates was used ^4^.

A study aiming to study the prevalence of HBV/HCV in hepatocellular carcinoma (HCC) patients in Gambia found that HBV carriage was present in 61% (129/211) of HCC while HCV present in 19% (36/191) of HCC patients ^32^. An AF of 50% for HBV and 21% for HCV obtained from sub-Saharan estimates was used ^33^. A study aiming to study the prevalence of H. pylori in Kano, Nigeria found a prevalence of 81.7% ^34^ while in Uganda 18 of the 21 cases of stomach cancer had H.pylori ^35^. However, an AF of 89% derived from world estimates was used ^4^ ( Table 2).

In South Africa, ova of *Schistosoma haematobium* were seen in microscopic sections of bladder tumours in 85% of the patients with squamous cell carcinoma, in 50% of those with undifferentiated tumours and adenocarcinoma, in 17% of those with mixed tumours or sarcoma, and in only 10% of the patients with transitional cell carcinoma (all classifications of the bladder tumours) ^36^. However, an AF of 41% was used derived from endemic areas in Africa ^4^.

We did not come across any studies in Africa showing the prevalence of *Opisthorchis viverrini* and *Clonorchis sinensis* in cancer of the bile duct. Similarly the AF could not be obtained. Burkitt’s lymphoma (BL) was first described in Eastern Africa where the highest incidence and mortality rates are seen. It has been associated with EBV and affects mainly children, where boys are more susceptible than girls ^31^. However, we did not come across any cases of BL or Adult T-cell leukaemia/lymphoma (ATLL) from our study.

## Results

### Demographics

An estimated total of 17,584 and 4304 cancer files were recorded in the KNH and MTRH respectively from 2008 to 2012. For the purpose of this study data was only obtained from 500 randomly selected files per hospital. In KNH, 60% of these were females while 40% were males with a mean age of 50.57 (18 to 95 years), giving a male to female (M:F) ratio of 1:1.5. In MTRH, 56% were females while 44% were males with a mean age of 48 years (18 to 90 years) giving a M:F ratio of 1:1.2. More than 70% of patients were within the age group of 25 to 64 years in both hospitals, largest age group being 45 to 64 years (43.4%) in KNH, and 25 to 44 years (39.4%) in MTRH. By Province, most KNH patients were from Central, Eastern and Western, (44%, 24.8% and 10.2% respectively), with specific regions being Muranga, Kiambu, Machakos, Nyeri and Kisii (11.2%, 10.2%, 8%, 8% and 5.4% respectively) with 66% of these patients being referred from other hospitals. On the other hand, most MTRH cancer patients were from the Rift valley, Western and Nyanza Provinces with 66.8%, 24.8% and 6.4% respectively with specific highest regions being Uasin Gishu, Nandi, Lugari, Trans Nzoia and Bungoma (22.8%, 8.4%, 8.4%, 7.4% and 6% respectively). 41% of these cancer cases were referrals from other hospitals ( Table 3).

**Table 3.** Cancer cases by age groups, gender, method of cancer diagnosis, region and referral information in KNH/MTRH: 2008–2012.

Site	KNH						MTRH					
Year	08	09	10	11	12	All	08	09	10	11	12	All
Age groups												
≤24 %	5 5.6	4 5	6 7.1	9 7.5	7 5.6	31 6.2	8 7.3	11 7.1	4 7	2 2.4	3 3.2	28 5.6
25–44 %	30 33.3	22 27.5	24 28.2	34 28.3	39 31.2	149 29.8	42 38.2	70 45.5	20 35.1	35 41.2	30 31.9	197 39.4
45–64 %	35 33.9	35 43.8	36 42.4	52 43.3	59 47.2	217 43.4	45 40.9	50 32.5	21 36.8	34 40	39 41.5	189 37.8
65–85 %	19 21.1	18 22.5	18 21.2	25 20.8	18 14.4	98 19.6	15 13.6	21 13.6	10 17.5	14 16.5	17 18.1	77 15.4
≥85 %	1 1.1	1 1.3	1 1.2	0 0	2 1.6	5 1	0 0	2 1.3	2 3.5	0 0	5 5.3	9 1.8
Total %	90 100	80 100	85 100	120 100	125 100	500 100	110 100	154 100	57 100	85 100	94 100	500 100
Gender												
Male %	39 43	37 46.3	33 38.8	51 42.5	40 32	200 40	50 45.5	74 48.1	17 29.8	25 29.4	52 55.3	218 43.6
Female %	51 56.7	43 53.7	52 61.2	69 57.5	85 68	300 60	60 54.5	80 51.9	40 70.2	60 70.6	42 44.7	282 56.4
Total %	90 100	80 100	85 100	120 100	125 100	500 100	110 100	154 100	57 100	85 100	94 100	500 100
Method of Cancer Diagnosis												
Radiology %	61 67.8	68 85	63 74.1	95 79.2	107 85.6	394 78.8	7 6.4	12 7.8	20 35.1	28 32.9	84 89.4	151 30.2
Biopsy %	90 100	80 100	85 100	120 100	125 100	500 100	110 100	154 100	57 100	85 100	94 100	500 100
Cancer Cases by Province												
Central %	40 44.4	30 37.5	37 43.5	57 47.5	56 44.8	220 44	1 0.9	1 0.6	1 1.8	3 3.5	1 1.1	7 1.4
Coast %	2 2.2	1 1.3	1 1.2	0 0	2 1.6	6 1.2	0 0	0 0	0 0	0 0	0 0	0 0
Eastern %	24 26.7	22 27.5	22 25.9	30 25	26 20.8	124 24.8	0 0	0 0	0 0	0 0	1 1.1	1 0.2
Nairobi %	2 2.2	1 1.3	3 3.5	6 5	8 6.4	20 4	0 0	0 0	0 0	0 0	0 0	0 0
Nyanza %	4 4.4	8 10	12 14.1	7 5.8	9 7.2	40 8	4 3.6	15 9.7	4 7.0	5 5.9	4 4.3	32 6.4
Rift Valley %	7 7.8	7 8.8	4 4.7	6 5	13 10.4	37 7.4	77 70	95 61.7	33 57.9	60 70.6	69 73.4	334 66.8
Western %	11 12.2	11 13.8	5 5.9	13 10.8	11 8.8	51 10.2	28 25.5	41 26.6	19 33.3	17 20	19 20.2	124 24.8
Others %	0 0	0 0	1 1.2	1 0.8	0 0	2 0.4	0 0	2 1.3	0 0	0 0	0 0	2 0.4
Total %	90 100	80 100	85 100	120 100	125 100	500 100	110 100	154 100	57 100	85 100	94 100	500 100
Referral												
Yes %	57 63.3	48 60	57 67.1	85 70.8	83 66.4	330 66	44 40	64 41.6	19 33.3	39 45.9	41 43.6	207 41.4
No %	33 36.7	32 40	28 32.9	35 29.2	42 33.6	170 34	66 60	90 58.4	38 66.7	46 54.1	53 56.4	293 58.6
Total %	90 100	80 100	85 100	120 100	125 100	500 100	110 100	154 100	57 100	85 100	94 100	500 100

% = percentage

### Types of cancers

**Figure 1.**
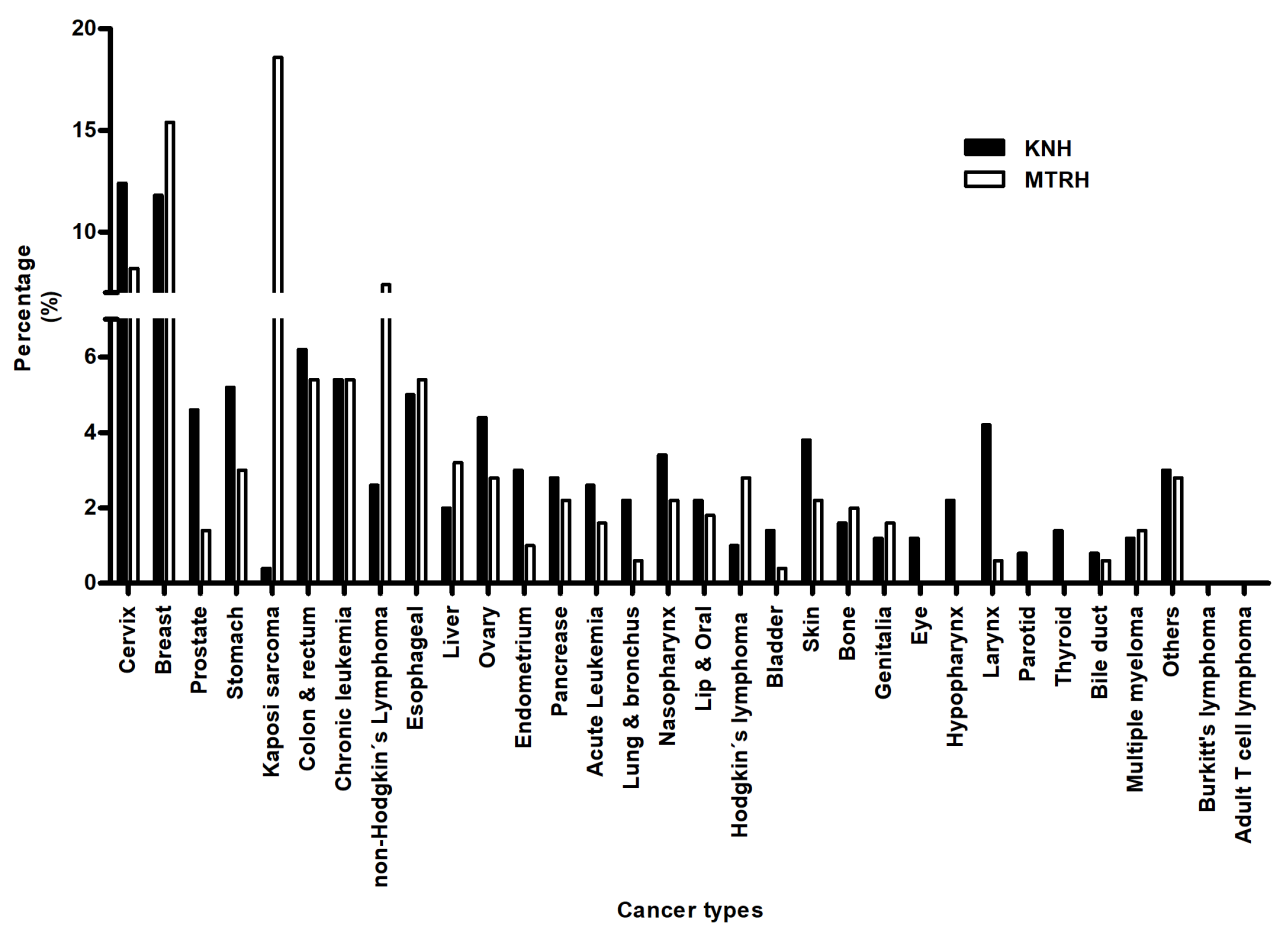
Types of cancers at Kenyatta National Hospital (KNH) and Moi Teaching and Referral Hospital (MTRH) from 2008–2012, Sample (500/500).

In KNH (n=500), the top five types of cancers were: cervical (62, 12.4%), breast (59, 11.8%), colorectal (31, 6.2%), chronic leukemia (27, 5.4%) and stomach cancers with 26 (5.2%). In females (n=300) the five most common cancers were cervical (62, 20.7%), breast (59, 19.7%), ovarian (22, 7.3%), chronic leukemia (16, 5.3%), and endometrial and stomach cancers both with 15 (5%). In males (n=200) the five most common types of cancers were prostate (23, 11.5%), laryngeal (19, 9.5%), colorectal (17, 8.5%), esophageal (14, 7.0%) and nasopharyngeal cancers with 12 (6.0%). In MTRH (n=500), the five most common types of cancer were Kaposi’s sarcoma (93, 18.6%), breast (77, 15.4%), cervical (41, 8.2%), non-Hodgkin’s lymphoma (37, 7.4%) and colorectal, chronic leukemia and esophagus cancer all with 27 (5.4%). In females (n=282) the five most common types of cancers were breast cancer (74, 26.2%), cervical (41, 14.5%), Kaposi’s sarcoma (38, 13.5%), non-Hodgkin’s lymphoma (15, 5.3%) and ovarian cancer with 14 (5%). In males (n=218) the five most common types of cancers were Kaposi’s sarcoma 55(25.2%), non-Hodgkin’s lymphoma 22(10.1%), chronic leukemia 17(7.8%), colorectal and esophageal cancer both with 16 (17.3%) ( Table 4 and Figure 1).

**Table 4.** Cancer cases by gender in Kenyatta National Hospital (KNH) and Moi Teaching and Referral Hospital (MTRH) from 2008–2012.

		KNH						MTRH					
Cancer site	ICD-0 code	No. of cases	P	Males		Females		No. of cases	P	Males		Females	
		N	%	n	%	n	%	N	%	n	%	N	%
Cervix uteri	C53	62	12.4	0	0	62	20.7	41	8.2	0	0	41	14.5
Breast	C50	59	11.8	0	0	59	19.7	77	15.4	3	1.4	74	26.2
Colon & Rectum	C18,C20	31	6.2	17	8.5	14	4.7	27	5.4	16	7.3	11	3.9
Chronic leukemia	C91.1,C92.1	27	5.4	11	5.5	16	5.3	27	5.4	17	7.8	10	3.5
Stomach	C16	26	5.2	11	5.5	15	5	15	3	5	2.3	10	3.5
Esophageal	C15	25	5	14	7	11	3.7	27	5.4	16	7.3	11	3.9
Prostate	C61	23	4.6	23	11.5	0	0	7	1.4	7	3.2	0	0
Ovary	C56	22	4.4	0	0	22	7.3	14	2.8	0	0	14	5
Larynx	C32	21	4.2	19	9.5	2	0.7	3	0.6	3	1.4	0	0
Skin	C44	19	3.8	11	5.5	8	2.7	11	2.2	7	3.2	4	1.4
Nasopharynx	C11	17	3.4	12	6	5	1.7	11	2.2	8	3.7	3	1.1
Endometrium	C54.1	15	3	0	0	15	5	5	1	0	0	5	1.8
Pancrease	C25	14	2.8	6	3	8	2.7	11	2.2	3	1.4	8	2.8
Acute leukemia	C91.0,C92.0	13	2.6	5	2.5	8	2.7	8	1.6	4	1.8	4	1.4
Non Hodgkin´s lymphoma	C82-C85, C96	13	2.6	7	3.5	6	2	37	7.4	22	10.1	15	5.3
Lung and bronchus	C34	11	2.2	8	4	3	1	3	0.6	1	0.5	2	0.7
Lip and oral cavity	C00-C14	11	2.2	6	3	5	1.7	9	1.8	4	1.8	5	1.8
Hypopharynx	C13	11	2.2	4	2	7	2.3	0	0	0	0	0	0
Liver	C22	10	2	5	2.5	5	1.7	16	3.2	13	5.9	3	1.1
Bone	C40-C41	8	1.6	6	3	2	0.7	10	2	7	3.2	3	1.1
Bladder	C67	7	1.4	5	2.5	2	0.7	2	0.4	1	0.5	1	0.4
Thyroid	C73	7	1.4	0	0	7	2.3	0	0	0	0	0	0
Genitalia	*	6	1.2	3	1.5	3	1	8	1.6	3	1.4	5	1.8
Eye	C69	6	1.2	3	1.5	3	1	0	0	0	0	0	0
Multiple myeloma	C90.0	6	1.2	4	2	2	0.7	7	1.4	5	2.3	2	0.7
Hodgkin´s lymphoma	C81	5	1	2	1	3	1	14	2.8	7	3.2	7	2.5
Parotid gland	C07	4	0.8	3	1.5	1	0.3	0	0	0	0	0	0
Bile duct	C22.1	4	0.8	3	1.5	1	0.3	3	0.6	1	0.5	2	0.7
Kaposi's sarcoma	C46	2	0.4	1	0.5	1	0.3	93	18.6	55	25.2	38	13.5
Others	**	15	3	11	5.5	4	1.3	14	2.8	10	4.6	4	1.4
Adult T cell Leukemia	C91.5	0	0	0	0	0	0	0	0	0	0	0	0
Burkitt´s Lymphoma	C83.7	0	0	0	0	0	0	0	0	0	0	0	0
**Total**		**500**	**100**	**200**	**100**	**300**	**100**	**500**	**100**	**218**	**100**	**282**	**100**

*Genitalia included cancer of the penis (C60), vagina (C52), vulva (C51), testis (C62) and pelvis (C63) while **others included cancers of renal (C65), head (C76), brain (C71), anus (C21), ear (C30), rhabdomyosarcoma (C49) and meninges (C70). N = number of cases P = percentage.

### Cancer cases by age groups

From the results generated from both hospitals, it was suggestive that some cancers were predominant in specific age groups. Acute leukemia, NHL, cancer of the bone, genitalia, HL and nasopharyngeal were predominant in the age group of 24 years and below. Cancer of the cervix was predominant in the age group of 22 to 44 years while breast and bile duct cancers were predominant between 45 to 64 years. The age-group of 65 to 84 years was predominated by esophagus, prostrate, larynx, endometrium and lip and oral cavity cancers. A larger age group ranging from 45 to 65 years was predominated by colorectal, stomach, ovary, lung and bronchus, liver, bladder and multiple myeloma cancers ( Table 5 and Table 6). This information is particularly important for estimating the age to go for the cancer checkups.

**Table 5.** Cancer cases by age groups in Kenyatta National Hospital (KNH) from 2008–2012.

KNH											
Cancer site	ICD-0 code	Age groups									
		≤24		24–44		45–64		65–84		≥85	
		**cases**	**%**	**cases**	**%**	**Cases**	**%**	**Cases**	**%**	**Cases**	**%**
Cervix uteri	C53	3	9.7	26	**17.4**	29	13.4	4	4.1	0	0
Breast	C50	2	6.5	19	12.8	30	**13.8**	8	8.2	0	0
Colon & Rectum	C18,C20	1	3.2	4	2.7	19	**8.8**	7	7.1	0	0
Chronic leukemia	C91.1,C92.1	1	3.2	12	**8.1**	10	4.6	4	4.1	0	0
Stomach	C16	1	3.2	3	2	14	**6.5**	6	6.1	2	40
Esophageal	C15	0	0	2	1.3	14	6.5	9	**9.2**	0	0
Prostate	C61	0	0	0	0	10	4.6	12	**12.2**	1	20
Ovary	C56	1	3.2	7	4.7	11	**5.1**	3	3.1	0	0
Larynx	C32	0	0	1	0.7	13	6	6	**6.1**	1	20
Skin	C44	2	**6.5**	9	6	4	1.8	4	4.1	0	0
Nasopharynx	C11	2	**6.5**	5	3.4	7	3.2	3	3.1	0	0
Endometrium	C54.1	0	0	4	2.7	6	2.8	5	**5.1**	0	0
Pancrease	C25	0	0	4	2.7	5	2.3	5	**5.1**	0	0
Acute leukemia	C91.0,C92.0	2	**6.5**	9	6	1	0.5	1	1	0	0
Non Hodgkin´s lymphoma	C82-C85, C96	3	**9.7**	5	3.4	4	1.8	1	1	0	0
Lung and bronchus	C34	0	0	4	2.7	6	**2.8**	1	1	0	0
Lip and oral cavity	C00-C14	1	3.2	4	2.7	1	0.5	5	**5.1**	0	0
Hypopharynx	C13	0	0	2	1.3	6	2.8	3	3.1	0	0
Liver	C22	0	0	3	2	6	**2.8**	1	1	0	0
Bone	C40-C41	4	**12.9**	3	2	1	0.5	0	0	0	0
Bladder	C67	0	0	3	2	1	0.5	3	**3.1**	0	0
Thyroid	C73	0	0	3	2	4	1.8	0	0	0	0
Genitalia	*	1	**3.2**	4	2.7	1	0.5	0	0	0	0
Eye	C69	1	3.2	3	2	1	0.5	1	1	0	0
Multiple myeloma	C90.0	0	0	1	0.7	4	**1.8**	0	0	1	20
Hodgkin´s lymphoma	C81	2	**6.5**	3	2	0	0	0	0	0	0
Parotid gland	C07	0	0	2	1.3	1	0.5	1	1	0	0
Bile duct	C22.1	0	0	0	0	2	**0.9**	2	2	0	0
Kaposi's sarcoma	C46	0	0	0	0	0	0	2	1	0	0
Others	**	4	12.9	4	2.7	6	2.8	1	1	0	0
Adult T cell Leukemia	C91.5	0	0	0	0	0	0	0	0	0	0
Burkitt´s Lymphoma	C83.7	0	0	0	0	0	0	0	0	0	0
**Total **		**31**	**100**	**149**	**100**	**217**	**100**	**98**	**100**	**5**	**100**

*Genitalia included cancer of the penis (C60), vagina (C52), vulva (C51), testis (C62) and pelvis (C63) while **others included cancers of renal (C65), head (C76), brain (C71), anus (C21), ear (C30), rhabdomyosarcoma (C49) and meninges (C70). N = number of cases P = percentage

**Table 6.** Cancer cases by age groups in Moi Teaching and Referral Hospital (MTRH) from 2008–2012.

MTRH											
Cancer site	ICD-0 code	Age groups									
		≤24		24–44		45–64		65–84		≥85	
		Cases	%	Cases	%	Cases	%	Cases	%	Cases	%
Cervix uteri	C53	0	0	20	**10.2**	16	8.5	4	5.2	1	11.1
Breast	C50	3	10.7	28	14.2	37	**19.6**	8	10.4	1	11.1
Colon & Rectum	C18,C20	0	0	9	4.6	10	5.3	7	**9.1**	1	11.1
Chronic leukemia	C91.1,C92.1	1	3.6	10	5.1	10	5.3	6	**7.8**	0	0
Stomach	C16	0	0	4	2	6	3.2	4	**5.2**	1	11.1
Esophageal	C15	0	0	3	1.5	15	7.9	8	**10.4**	1	11.1
Prostate	C61	0	0	0	0	2	1.1	4	**5.2**	1	11.1
Ovary	C56	0	0	6	3	4	2.1	4	**5.2**	0	0
Larynx	C32	0	0	1	0.5	1	0.5	1	**1.3**	0	0
Skin	C44	1	3.6	2	1	3	1.6	4	**5.2**	1	11.1
Nasopharynx	C11	2	**7.1**	5	2.5	3	1.6	1	1.3	0	0
Endometrium	C54.1	0	0	0	0	2	1.1	3	**3.9**	0	0
Pancrease	C25	1	**3.6**	2	1.0	6	3.2	0	0	2	**22.2**
Acute leukemia	C91.0,C92.0	2	**7.1**	4	2.0	1	0.5	1	1.3	0	0
Non Hodgkin´s lymphoma	C82-C85, C96	6	**21.4**	9	4.6	17	9	5	6.5	0	0
Lung and bronchus	C34	0	0	0	0	1	0.5	2	**2.6**	0	0
Lip and oral cavity	C00-C14	1	3.6	3	1.5	1	0.5	4	**5.2**	0	0
Hypopharynx	C13	0	0	0	0	0	0	0	0	0	0
Liver	C22	1	3.6	6	3	5	2.6	4	**5.2**	0	0
Bone	C40-C41	2	**7.1**	5	2.5	2	1.1	1	1.3	0	0
Bladder	C67	0	0	0	0	2	**1.1**	0	0	0	0
Thyroid	C73	0	0	0	0	0	0	0	0	0	0
Genitalia	*	1	**3.6**	6	3	1	0.5	0	0	0	0
Eye	C69	0	0	0	0	0	0	0	0	0	0
Multiple myeloma	C90.0	0	0	0	0	5	**2.6**	2	**2.6**	0	0
Hodgkin´s lymphoma	C81	3	**10.7**	7	3.6	3	1.6	1	1.3	0	0
Parotid gland	C07	0	0	0	0	0	0	0	0	0	0
Bile duct	C22.1	0	0	1	0.5	2	**1.1**	0	0	0	0
Kaposi's sarcoma	C46	4	14.3	61	31	27	14.3	1	1.3	0	0
Others	**	0	0	5	2.5	7	3.7	2	2.6	0	0
Adult T cell Leukemia	C91.5	0	0	0	0	0	0	0	0	0	0
Burkitt´s Lymphoma	C83.7	0	0	0	0	0	0	0	0	0	0
**Total **		**28**	**100**	**197**	**100**	**189**	**100**	**77**	**100**	**9**	**100**

*Genitalia included cancer of the penis (C60), vagina (C52), vulva (C51), testis (C62) and pelvis (C63) while **others included cancers of renal (C65), head (C76), brain (C71), anus (C21), ear (C30), rhabdomyosarcoma (C49) and meninges (C70). N = number of cases P = percentage

### Infection-attributable cancers

The cancers listed here are cancers associated with the 11 infectious agents as classified and established by the International Agency for Research on Cancer (IARC). In KNH, 154 (30.8%) of the total cancers sampled were associated with infectious agents, while an estimated 138 (27.6%) were attributable to infections. Cancers of the cervix (62, 12.4%), stomach (26, 5.2%), nasopharynx (17, 3.4%), non-Hodgkin’s lymphoma (13, 2.6%) and liver (10, 2%) were the commonest infection-associated cancers. In MTRH, 241 (48.2%) of the 500 cancers cases sampled were associated with infectious agents, while an estimated 222 (44.4%) were attributable to infections. Kaposi’s sarcoma (93, 18.6%), cancer of the cervix (41, 8.2%) and non-Hodgkin’s lymphoma (37, 7.4%), liver (16, 3.2%) and stomach (15, 3%) were the commonest infection-associated cancers ( Table 7).

**Table 7.** Attributable fraction (AF) and estimated number of cancers attributable to infectious agents in KNH/MTRH from 2008–2012.

Infectious agent	Cancer site	ICD-0 code	AF%	No. of cancer cases KNH	No. of cancer cases attributable to infections KNH	No. of cancer cases MTRH	No. of cancer cases attributable to infections MTRH	No. of cancer cases attributable to infections KNH&MTRH
**Female**								
HPV	Cervix	C53	100	62	62	41	41	103
	Vulva	C51	40	2	1	3	1	2
	Vagina	C52	78	1	1	0	0	1
	Anus	C21	88	2	2	0	0	2
	Oropharynx	C10	31	0	0	0	0	0
*Schistosoma*	Bladder	C67	41	2	1	1	0	1
HBV/HCV	Liver	C22.0	71	5	4	3	2	6
EBV	Nasopharynx	C11	96	5	5	3	3	8
	HL	**C81**	74	3	2	7	5	7
*H. pylori*	Stomach	C16	89	15	13	10	9	22
EBV/HIV	NHL	**C82-85,C96**	100	6	6	15	15	21
HIV/HHV8	KS	**C46**	100	1	1	38	38	39
*O. viverrini* *C. sinensis*	Bile duct	C22.1	*	1	**	2	**	
Total (% female cancers)				105(35.0%)	98 (32.7%)	123(43.6%)	114 (40.4%)	212 (36.4%)
**Male**								
HPV	Penis	C60	50	0	0	2	1	1
	Anus	C21	88	3	3	4	4	7
EBV	Oropharynx	C10	31	0	0	0	0	0
*Schistosoma*	Bladder	C67	41	5	2	1	0	2
HBV/HCV	Liver	C22.0	71	5	4	13	9	13
EBV	Nasopharynx	C11	96	12	12	8	8	20
	HL	**C81**	74	2	1	7	5	6
*H. pylori*	Stomach	C16	89	11	10	5	4	14
EBV/HIV	NHL	**C82-85,C96**	100	7	7	22	22	29
HIV/HHV8	KS	**C46**	100	1	1	55	55	56
*O. viverrini* *C. sinensis*	Bile duct	C22.1	*	3	**	1	**	0
Total (% of male cancers)				49 24.5%	40 20%	118 54.1%	108 49.5%	148 (35.41%)
**Total (% of cancers in both sexes)**				154 30.8%	138 27.6%	241 48.2%	222 44.4%	360 36%

Over the 5-year period the total number of cases sampled in each hospital were 500. KNH had 300 female and 200 male cases while MTRH had 282 female and 218 male cases. *AF not available, ** Not possible to estimate without AF,

## Discussion

In females (n=300) the five most common cancers in KNH were cervical, breast, ovarian, chronic leukemia, endometrial and stomach while in MTRH (n=282), they were breast, cervical, Kaposi’s sarcoma, non-Hodgkin’s lymphoma and cancer of the ovary. These results are comparable with the data obtained from a previous study conducted retrospectively in Tenwek Hospital, in Bomet District, western Kenya in the period of 1999 to 2007 that showed that the common types of cancer in women were cervical, breast, stomach, uterus and esophageal ^37^. Similarly an incidence rates study done using the Nairobi cancer registry data found breast, cervical, esophageal, large bowel stomach and ovarian cancers to be the most incident ^38^. The high number of cervical cancer could reflect a potential higher prevalence of HPV infection, low screening rates or late detection of the disease. Most patients with cervical cancer were between in the age groups of 24 to 44 and 45 to 54 years while for breast cancer, they were in the age group of 45 to 64 years followed by 24 to 44 years with 35.5% and 29% respectively. The risk factors associated with breast cancer include early menarche, late childbearing, having fewer children, obesity, lack of awareness and early detection ^39^.

In males, (n=200) the five most common types of cancers in KNH were prostate, laryngeal, colorectal, esophageal and nasopharyngeal carcinoma while in MTRH, (n=218) they were: Kaposi’s sarcoma, non-Hodgkin’s lymphoma, chronic leukemia, colorectal and esophageal cancers. The high number of prostate and esophageal cancer is comparable with the data from the Kenya National cancer control strategy that showed that Kaposi’s sarcoma, prostate and esophageal cancer to be the most common cancers in men ^40^. Similarly the retrospective study done in Tenwek Hospital in Western Kenya from 1999 to 2007 showed that the most common cancers in men were esophagus, stomach, prostate and colorectal and non-Hodgkin’s lymphoma (NHL) ^37^. Similarly an incidence rates study using the Nairobi cancer registry data found prostate, esophageal, large bowel, stomach, oral and liver cancers to be the most incident ^38^. Other studies show that esophageal is the leading cause of death among both men and women in East Africa ^37, 39, 41^. The majority of the esophageal cancers were in patients aged 65 to 84 years, followed by 45 to 64 years and was more common in males than women. Some of the risk factors independently associated with esophageal cancer ( *P* < 0.05) identified from a study conducted at MTRH were low socio-economic status, smoking, alcohol consumption, tooth loss, cooking with charcoal and firewood, consumption of hot beverage and use of a traditional fermented milk referred to as *mursik* ^41^.

Elsewhere, a study aiming to determine the burden and pattern of cancer in Western Kenya by use of data from the Eldoret cancer registry, from 1999 to 2006, found out that about 21% of the patients had haematological malignancies where lymphomas were the most common (11.9%) followed by acute and chronic leukemia with 4.0% and 3.2% respectively. Esophageal (10.5%), breast (6.2%) and Kaposi’s sarcoma (5.9%) were the top most non-haematological cancers. From our study, KS, NHL and chronic leukemia were high especially in MTRH ^42^. Chronic leukemia was specifically the 4 ^th^ most common type of cancer in KNH and 5 ^th^ most common in MTRH while acute leukemia was also high and highest in the age-group of 24 years and below. It was suggestive that prostate cancer commonly affected males in the age-group of 65 to 84 years in both hospitals and with notable higher occurrence also in the age-group of 45 to 64 years. These differences could possibly be attributed to lifestyle choices and family history of the disease which are among the risk factors associated elsewhere with the cancer ^43^.

Infection-attributable cancers contributed up to a total of 36% of the total cancer burden in both hospitals. Specifically this was 27.6% for KNH and 44.4% for MTRH. This is closer to estimates of 31.1% for sub-Saharan Africa; higher than 9.2% estimated for developed countries and expectedly higher than global average of 15.4% reported by Plummer *et al.* in 2016 ^4^. Fortunately, the majority of cancers associated with infectious agents can be controlled by controlling the infectious agent ^14, 44^. Cancers of the cervix, stomach, nasopharynx, liver and NHL were the commonest infection-associated cancers in KNH; while in MTRH, KS, cancer of the cervix, NHL, liver and stomach cancer were the commonest infection-associated cancers. The high burden of cancers of the cervix, stomach and liver are comparable with other population based studies that show that among infection-related cancers, stomach, liver and cervical cancer, not only account for the vast majority of the total cancer burden associated with infections, but they have the highest incidences ^4, 14, 45^. Kaposi´s sarcoma, non-Hodgkin´s lymphoma and cervical cancer are HIV-associated malignancies ^5, 7^. The high numbers of Kaposi´s sarcoma, non-Hodgkin´s lymphoma and cervical cancer at MTRH could have been influenced by the source of data that was obtained from the AMPATH-Oncology centre that evolved from an existing HIV program ^9^. The information on the HIV status of the patients from whose files were used, was not obtained to accurately link the associations. In Kenya, a HIV prevalence of 5.6% (95% CI: 4.9 to 6.3), and HIV incidence of 0.5% (95% CI: 0.2 to 0.9), corresponding to an annual HIV transmission rate of 8.9 per 100 HIV-infected persons has been reported ^6^. This HIV pandemic could have influenced the high number of HIV-associated malignancies as documented by 4, 7, 46.

A high number of liver cancer cases were observed in males rather than females. This can be explained by higher prevalence of risk factors for liver cancer in men as compared to women such as higher alcohol consumption and infection with HBV or HCV ^14^. The high number of gastric cancer in KNH suggested a higher prevalence of the risk factors associated with the cancer including infection with *H. pylori,* poor diet, poor hygiene, lack of awareness or the late stage of diagnosis ^14, 47^.

More than 70% of patients were 25 to 64 years of age in both hospitals with the highest age group being 45 to 64 years in KNH (43.4%) and 25 to 44 years in MTRH (39.4%). According to the latest data published in WHO 2018, life expectancy in Kenya is 64.4 years for males and 64.8 for females the age frequently associated with cancer occurrence ^48^. There were more female cases than males with 60% and 56.4% in KNH and MTRH respectively, this could be explained by the fact that probably males fear going to the hospitals, or women tend to have frequent contact with the health professionals and show up in even greater numbers than men during health campaigns. The findings of the study show a high number of referrals at KNH (66%) as compared to the 41.4% at MTRH. The possible reasons for the high referral rates at KNH could be explained by the establishment of a functional oncology centre which encourages the referral of patients for care while the decline at MTRH could be due to the establishment of an outreach satellite oncology service sites based at Eldoret where a significant number of patients are attended without the need of going to MTRH ^42^. The distant referrals was an indication that majority of the patients have difficulty accessing cancer services.

## Limitations

Our study had several limitations. First, causality could not be proven and we could not ascertain that a given cancer was actually caused by an infectious agent including the high number of HIV-associated malignancies at MTRH. Therefore, we used AF generated from other studies. Information on associations is sparse in Kenya which opens up new avenues for future research studies. Our study used a sampling faction of the total number of files available at the two hospitals as compared to studying all the files that could have influenced our results. For failure of not knowing the whole sampling frame, there was a probability of introducing selection bias by the use of convenient sampling method to select the files at MTRH. This could result in over or under-representation of the cases. Future studies should focus more on population based data or use proportions of individual cancers to calculate the sample size. Population based data would be more reflective of the overall population. Our choice of study population was influenced by the fact that cancer registration in Kenya was in its fairly early stages of development at the time the study was done, and the use of hospital based data seemed suitable. The percentage attributable to infections for certain cancers, such as BL, adult T-cell leukemia or bile duct cancers could not be calculated because we did not come across any cases of the first two cancers while the AF of bile duct was unobtainable.

## Conclusion

Our study presented a picture of the burden of cancer and infection-attributable cancer from a hospital point of view. Despite the limitations, the role played by infectious agents in contributing to the overall cancer burden was highlighted. Controlling for the infectious agents could translate to a significant reduction in the cancer burden. Further research is warranted to prove causality between infection-attributable cancers and the infectious agents in Kenya as this may provide new avenues for effective cancer prevention.

## Data availability

The data underlying this study is available from the Open Science Framework (OSF) Dataset 1: Burden of Cancer in Kenya; Types, Infection-Attributable and Trends: A National Referral Hospital Retrospective Survey https://doi.org/10.17605/OSF.IO/MD2PY ^49^

Data is available under a CC0 1.0 universal license.

## Supplementary material

**Supplementary File 1: Data collection form**
