# Supplementary material for: Cancer in Kenya: types and infection-attributable. Data from the adult population of two National referral hospitals (2008-2012)
Source: AAS Open Res. 2019 Nov 14;1:25. Originally published 2018 Sep 25. [Version 5] doi: 10.12688/aasopenres.12910.5 (PMC7185250; doi:10.12688/aasopenres.12910.5)
Supplement: Supplementary file 1 [file aasopenres-1-14118-s0000.tgz › e278b1e2-f4b0-44fd-99fc-f699953673ad_s2_questionnaire.docx]

S1 APPENDIX: DATA COLLECTION FORM

TOPIC: **BURDEN OF CANCER ASSSOCIATED WITH INFECTIOUS AGENTS AT FOUR REFERRAL HOSPITALS, KENYA**

QUESTIONNAIRE NUMBER: …………………………….

**SITE**

1. Kenyatta National hospital (KNH)
2. Moi Teaching and Referral Hospital (MTRH)
3. Coast Province General Hospital
4. Jaramogi Oginga Odinga Teaching and Referral Hospital

**PATIENT GENERAL INFORMATION**

Age of patient………………………….

Sex Male

Female

Patient’s origin (place of birth)…………………………

Was the patient a referral?

Yes No

If so specify…………………………………..

**PATIENT MEDICAL HISTORY**

1. **Type of cancer**

List of common types of cancers

Gastric cancer Breast cancer

Cervical cancer colorectal cancer

Liver cancer Lung and Bronchus cancer

Esophageal Nasopharyngeal carcinoma

Burkitt’s lymphoma Lip and Oral cavity

Hodgkin’s lymphoma Bladder cancer

Non Hodgkin’s lymphoma Prostate cancer

Adult T cell leukemia Kaposi Sarcoma

Leukemia Skin cancer

Osteogenic Pancrease

Ovary Endometrium

Eye Hypopharyngeal

Laryngeal Parotid

Thyroid Cholangiocarcinoma

Genitalia (Penis, Vaginal, Vulva, Testis)

Multiple myeloma

OTHERS Specify………………………………

1. **Method of cancer diagnosis used**.

1. **Biopsy**

2. **Aspiration** 2.1.Fine needle aspiration

2.2. Bone marrow aspiration

3. **Blood test**

4. **Pap smear**

5. **Radiological/Imaging**

5.1. X-ray

5.2. Computed Tomography (CT)

5.3. Magnetic Resonance Imaging (MRI)

5.4. Ultrasound

5.5. Mammogram

OTHERS Specify………………………………

1. **Year of diagnosis**

Year 2008

Year 2009

Year 2010

Year 2011

Year 2012
