# Supplementary material for: Cancer in Kenya: types and infection-attributable. Data from the adult population of two National referral hospitals (2008-2012)
Source: AAS Open Res. 2019 Nov 14;1:25. Originally published 2018 Sep 25. [Version 5] doi: 10.12688/aasopenres.12910.5 (PMC7185250; doi:10.12688/aasopenres.12910.5)
Supplement: Peer review history

# Peer review: Reviewer response for version 5

**DOI:** 10.21956/aasopenres.14118.r27260

**Published:** 2019-11-27

**Authors:** Delphine Casabonne

**Affiliations:** 1 Unit of Infections and Cancer, Cancer Epidemiology Research Programme, Catalan Institute of Oncology, Barcelona, Spain; 2 Epidemiology and Public Health Networking Biomedical Research Centre (CIBERESP), Madrid, Spain

The issue on the data collection and availability of the HIV status of the patients should be also mentioned in the Methods.

 The sentence below in the overall Discussion is not clear:

 "The information on the HIV status of the patients from whose files were used, was not obtained to accurately link the associations." 

 Could you rephrase it? Here is a suggestion:

 "Unfortunately, the information on the HIV status of the patients from whose files were used was not collected and therefore it was not possible to accurately link a cancer site to HIV infection."

I confirm that I have read this submission and believe that I have an appropriate level of expertise to confirm that it is of an acceptable scientific standard.

# Peer review: Reviewer response for version 4

**DOI:** 10.21956/aasopenres.14096.r27194

**Published:** 2019-10-25

**Authors:** Delphine Casabonne

**Affiliations:** 1 Unit of Infections and Cancer, Cancer Epidemiology Research Programme, Catalan Institute of Oncology, Barcelona, Spain; 2 Epidemiology and Public Health Networking Biomedical Research Centre (CIBERESP), Madrid, Spain

The authors addressed most of the issues. However, further points should be corrected and/or specified:

Main comments:

1. The authors mentioned that " *The files for 2008 to 2011 were obtained from the oncology centre at the Academic Model for the Prevention and Treatment of HIV/ AIDS (AMPATH)*". The authors should clarify if all patients selected in this center for these years (81% of the sample for MTRH) were HIV+ or the authors should report if the HIV status of these patients was unknown. This information should be added to the manuscript to help interpreting the higher and different cancer proportions in MTRH and KNH.
2. In Table 2 (Previous prevalence studies on infectious agents and cancers): The virus HTLV should be associated with T-cell lymphoma and not Burkitt lymphoma. Burkitt lymphoma should be associated with EBV.
3. Table 6 (Cancer cases by age groups in Moi Teaching and Referral Hospital (MTRH) from 2008–2012) should be checked again. For instance the total number of KS across the age for MTRH is not correct (N=2) and it should be N=93. The authors should check the tables again.

Minor comments:

1. In the abstract: Replace "stomach cancer 26 (5.2%)" by "stomach cancer (26, 5.2%)".

I confirm that I have read this submission and believe that I have an appropriate level of expertise to confirm that it is of an acceptable scientific standard, however I have significant reservations, as outlined above.

# Response

**Published:** 2019-11-02

**Authors:** Lucy Wanjiku Macharia

**Affiliations:** University of Nairobi (Alumni), Kenya

Dear Dr. Delphine Casabonne,

The authors are thankful to you for reviewing our manuscript and for the suggestions. We have modified the manuscript and double-checked the tables to incorporate the suggestions. Kindly find below the point-by-point response to the queries.

**The authors mentioned that " *The files for 2008 to 2011 were obtained from the oncology centre at the Academic Model for the Prevention and Treatment of HIV/ AIDS (AMPATH)*". The authors should clarify if all patients selected in this center for these years (81% of the sample for MTRH) were HIV+ or the authors should report if the HIV status of these patients was unknown. This information should be added to the manuscript to help interpreting the higher and different cancer proportions in MTRH and KNH.**

We thank the reviewer for the question and the suggestion. We do not know the HIV information of the patients whose files were obtained from AMPATH.  We have highlighted the suggestion in the discussion and limitations section.

**In Table 2 (Previous prevalence studies on infectious agents and cancers): The virus HTLV should be associated with T-cell lymphoma and not Burkitt lymphoma. Burkitt lymphoma should be associated with EBV.**

We agree with the reviewer’s observation. We have modified Table 2 and corrected the misclassification. We are thankful for the observation.

**Table 6 (Cancer cases by age groups in Moi Teaching and Referral Hospital (MTRH) from**

**2008–2012) should be checked again. For instance the total number of KS across the age**

**for MTRH is not correct (N=2) and it should be N=93. The authors should check the tables again.**

We are grateful to the reviewer for the keen observation. We have checked the tables and made the necessary modifications to Tables 5 and 6. We are thankful for the suggestion.

**Minor comments:**

**In the abstract: Replace "stomach cancer 26 (5.2%)" by "stomach cancer (26, 5.2%)".**

We thank the reviewer for the observation. We have modified the statement highlighted above in the abstract section.

# Peer review: Reviewer response for version 3

**DOI:** 10.21956/aasopenres.14030.r27129

**Published:** 2019-08-30

**Authors:** Kalina Duncan

**Affiliations:** 1 Center for Global Health, U.S. National Cancer Institute, Rockville, MD, USA

In the methods section: You note there are "few" radiation machines but more may be coming. It would help the context here to report the number and perhaps remove the bit about more coming unless it can be cited in some way? Or if you have provide more specificity about how and where those machines are intended to go, that will be helpful. 

 Also in Methods: you introduce AMPATH without clarifying what it is. I would recommend providing a sentance of background of their role at MTRH.

 In Discussion: At KNH, there is a variability in breast cancer proportions that may be interesting to look at. From 2008-2011, the proportion was between 10-15%. In 2012, it drops to below 5%. What happened that year? Were there really fewer cases? Was there an issue with data quality (more incomplete records, etc.)? 

 In Discussion: You have this line: "The high number of Kaposi's sarcoma, non-Hodgkin's lymphoma and cervical cancer could have been influenced by the association of the cancers with HIV" regarding the high number of HIV-related cases at MTRH. I think this actually requires further discussion, as it pushes the infection-related rates way above average. The presence of the AMPATH program, and its specialization in treating patients with HIV should be noted in discussion, as explanation. Because these are hospital-based data, this is especially important to note as this hospital attracts many HIV positive patients, most likely. 

 I would consider an examination and a citation to the article listed below, "Viral-associated malignancies in Africa: are viruses ‘infectious traces’ or ‘dominant drivers’?" ^1^.

I confirm that I have read this submission and believe that I have an appropriate level of expertise to confirm that it is of an acceptable scientific standard.

## References

[1] : Viral-associated malignancies in Africa: are viruses 'infectious traces' or 'dominant drivers'?. *Curr Opin Virol*.20: 10.1016/j.coviro.2016.08.002 28-33 10.1016/j.coviro.2016.08.002 27551983

# Response

**Published:** 2019-10-03

**Authors:** Lucy Wanjiku Macharia

**Affiliations:** University of Nairobi (Alumni), Kenya

**Dear Dr. Kalina Duncan,**

 Thank you for reading our manuscript, for the suggestions and the approval status.  Kindly find below the response to the queries.

**Comments**

**In the methods section: You note there are "few" radiation machines but more may be coming. It would help the context here to report the number and perhaps remove the bit about more coming unless it can be cited in some way? Or if you have provide more specificity about how and where those machines are intended to go, that will be helpful.**

 We thank the reviewer for the comment. We have edited the statement, included the number of radiation machines and removed “the more are coming” bit due to lack of a proper citation.

**Also in Methods: you introduce AMPATH without clarifying what it is. I would recommend providing a sentence of background of their role at MTRH.**

 We are thankful for the suggestion. We have included a sentence to connect AMPATH and MTRH.

**In Discussion: At KNH, there is variability in breast cancer proportions that may be interesting to look at. From 2008-2011, the proportion was between 10-15%. In 2012, it drops to below 5%. What happened that year? Were there really fewer cases? Was there an issue with data quality (more incomplete records, etc.)?**

 We agree with the reviewer’s concern.  The variability from the proportions of the breast cancer at KNH could have been influenced by various factors including the sample size used as compared to sampling the whole frame which was not feasible due to cost constraints. Similarly, maybe there were high numbers of patients with breast cancer that visited the hospital in certain years as compared to others. We have removed the proportion section from the results and discussion in line too with the suggestion from the previous reviewer. We are thankful for the keen observation.

**In Discussion:** **You have this line: "The high number of Kaposi's sarcoma, non-Hodgkin's lymphoma and cervical cancer could have been influenced by the association of the cancers with HIV" regarding the high number of HIV-related cases at MTRH. I think this actually requires further discussion, as it pushes the infection-related rates way above average . The presence of the AMPATH program, and its specialization in treating patients with HIV should be noted in discussion, as explanation. Because these are hospital-based data, this is especially important to note as this hospital attracts many HIV positive patients, most likely. I would consider an examination and a citation to the article listed below, "Viral-associated malignancies in Africa: are viruses ‘infectious traces’ or ‘dominant drivers’?".**

**References**

**1. Rochford R, Korir A, Newton R: Viral-associated malignancies in Africa: are viruses 'infectious traces' or 'dominant drivers'?. *Curr Opin Virol*. 20: 28-33 PubMed**

 We thank the reviewer for the suggestion. We have modified the discussion section to incorporate the new suggestions and reference.

# Peer review: Reviewer response for version 3

**DOI:** 10.21956/aasopenres.14030.r26842

**Published:** 2019-04-17

**Authors:** Delphine Casabonne

**Affiliations:** 1 Unit of Infections and Cancer, Cancer Epidemiology Research Programme, Catalan Institute of Oncology, Barcelona, Spain; 2 Epidemiology and Public Health Networking Biomedical Research Centre (CIBERESP), Madrid, Spain

The authors selected 500 (partially randomly selected) files of cancer patients from two National referral hospitals in Kenya and reported the cancer prevalence. The authors then applied attributable fraction (AF) for infection-associated cancers from published literature (meta-analysis, prevalence data and relative risk estimates from previous studies and WHO report). The authors estimated that 28% and 44% of the observed cancers were attributable to infections in KNM and MTRH hospitals, respectively.

**Title:**

- Please add in the title the words “adult” and the years “2008-2012”.

 

**Abstract:**

- Please indicate in the abstract and methods not only the selected years (2008-2012) but also the months.

 

**Methods:**

- Please indicate in the abstract and methods not only the selected years (2008-2012) but also the months.
- *Sample size calculation*: This is a case-study and the prevalence of cancers is not the prevalence of cancers in Kenya but the proportion of a specific cancer subtypes within the total number of cancer cases in the hospitals. Proportions estimated in Kenya in different published reports could have been used. For example, in https://gco.iarc.fr/today, the cancer prevalence by subtypes in Kenya for 2018 were estimated to be: breast: 13.6%, cervix: 11.5%, oesophagus: 8.4%, prostate: 5.8%, colorectum: 4.8%, etc. It might therefore be more informative to give the number of patients needed from different cancer subtype proportions (p=0.01, 0.05, 0.10, 0.15 and 0.20).
- The details of the calculations are not needed in the text. Please delete these two sections of the text (page 4):
  a) “Therefore, n=1.96^2...” to “n=384”.
  b)  In the text from “N1= 3168...” to “... ∑ni=500”. This information is provided in Table 1.
- More details should be given in relation to the selection of the records. What is the “non-response allowance” in this cross-sectional design? Were all 500 selected records available for both hospitals? If a selected record had missing data (based on the pre-designed questionnaire) was it replaced by another randomly selected record? This part is unclear; please clarify.
- For MTRH, the origin of the 2008-2011 files is unclear. Which “convenient sampling” was used for the 390 files of MTRH? Could the authors explain better the procedure of selection for this hospital? How could biases be introduced for interpretability? How is representativeness kept?
- Please modify the methods section accordingly.

**Results:**

- As mentioned by previous reviewers, the higher prevalence of KS and NHL in MTRH might reflect the tight relationship of this hospital with AMPATH/HIV. Also, the convenience sampling method used for the selection of the records for this hospital might accentuate the number of KS and NHL cases observed in this hospital. Please add to the current Table 7 the estimates of the 2 hospitals altogether and mention the overall 36% of cancers attributable to infections agents.
- *Figures 2 & 3*: The titles and the figures are unclear and inconsistent with the text. From the results section, in KNH hospital, the first commonest cancers in males were prostate (11.5%) and laryngeal (9.5%) cancers. Why is colorectal cancer plotted in Figure 2? In page 9, why do the authors mention access to treatment in relation to the histograms in the result section? Also, the different units in the y-axis are misleading. Finally, due to the small sample size by year and the use of convenience sampling for MTRH, I would suggest not to show Figures 2 and 3. Please delete the text in the results section associated with these Figures (page 9, “Proportions of the top most cancer cases”).

*Minor comments*:

- Since penile cancer is one of the cancer sites associated with infections please provide the number of cases of penile cancers that were observed in each hospital. If none were observed please provide this information in the text.
- The authors mentioned 11 infections in page 9 but there are less infections in Table 2. Please modify accordingly.
- Can the referral hospital for KNH be MTRH? And vice versa?

**Discussion:**

- As mentioned by previous reviewers, the authors gave too much emphasis on comparing the two hospital results. The presence of KS and NHL in the top four cancers for MTRH should be put in perspective with the hospital origin of the patients (HIV center). The authors should discuss this point in more detail. Given the method of selection of the patients, KNH might reflect better the overall prevalence.
- In the limitations, the representativeness of the selected patients should be addressed. How complete are the hospital registration of the patients? Could any biases be introduced at this stage? Are some cancer subtypes less likely to be recorded in these hospitals? Is misdiagnosis a potential issue?

*Minor comments:*

- In page 13, “Similarly an incidence using the Nairobi cancer registry...” , please correct this sentence. Do you mean incidence rates?
- Please delete all references to the result part (Tables X, figures Y, ...) in the Discussion section.

**Summary:**

- This article describes the cancer prevalence and cancers attributable to infections in two hospitals from Kenya. Due to the selection methods there are important issues to take into account when interpreting the results - in particular in relation to HIV-associated cancers - that have not been addressed adequately.

I confirm that I have read this submission and believe that I have an appropriate level of expertise to confirm that it is of an acceptable scientific standard, however I have significant reservations, as outlined above.

# Response

**Published:** 2019-10-03

**Authors:** Lucy Wanjiku Macharia

**Affiliations:** University of Nairobi (Alumni), Kenya

**Dear Dr. Delphine Casabonne,**

 We are thankful to you for reading our manuscript, for the positive criticism and the suggestions. Kindly find below the answers to the questions highlighted.

**Title:**

**Please add in the title the words “adult” and the years “2008-2012”.**

 We have added the words “adult” and years to the title. Thank you for the suggestion.

**Abstract:**

**Please indicate in the abstract and methods not only the selected years (2008-2012) but also the months.**

 We have modified the abstract and method section to include the years and months. We are grateful for the suggestion.

**Methods:**

**Please indicate in the abstract and methods not only the selected years (2008-2012) but also the months**.

 We have included the new suggestions to the abstract and methods section.

***Sample size calculation*** **: This is a case-study and the prevalence of cancers is not the prevalence of cancers in Kenya but the proportion of a specific cancer subtypes within the total number of cancer cases in the hospitals. Proportions estimated in Kenya in different published reports could have been used. For example, in https://gco.iarc.fr/today, the cancer prevalence by subtypes in Kenya for 2018 were estimated to be: breast: 13.6%, cervix: 11.5%, oesophagus: 8.4%, prostate: 5.8%, colorectum: 4.8%, etc. It might therefore be more informative to give the number of patients needed from different cancer subtype proportions (p=0.01, 0.05, 0.10, 0.15 and 0.20).**

 We are thankful for the comment. Our objective was to sample 500 files from each hospital and identify how many cancer types were in the 500 selected files as a representative sample. This is because we could not afford to sample the whole population. For this, the *p *we used in the sample size calculation was *p* for expected prevalence or proportion or estimated proportion of a disease (Cancer) as explained by references (9 to 11) which we assumed a p of 50% for not knowing the prevalence at the time which is also acceptable as explained by Arya et. al., 2012 (Reference 12). This is the sample size that was also approved by the ethical committees.  To apply the proportions of individual cancer type, we would need to know the information on the total numbers of the specific cancers in both the hospitals, information that was unavailable.

 Lastly, as we cannot change the sample size calculation strategy at this stage, we can only highlight how we arrived at the sample size of 500 files and highlight the new suggestion and a potential limitation. We are thankful for the suggestion.

**The details of the calculations are not needed in the text. Please delete these two sections of the text (page 4):**

**a) “Therefore, n=1.96^2...” to “n=384”.**

**b) In the text from “N1= 3168...” to “... Σni=500”. This information is provided in Table 1.**

 The calculation details highlighted above have been removed from the text. We are thankful for the suggestion

**More details should be given in relation to the selection of the records. What is the “non-response allowance” in this cross-sectional design?**

 We have inserted a statement to clarify the meaning of the “non-response allowance” in the context of our study. We appreciate the comment.

**Were all 500 selected records available for both hospitals? If a selected record had missing data (based on the pre-designed questionnaire) was it replaced by another randomly selected record? This part is unclear; please clarify.**

 Yes. All the targeted 500 files were available from both hospitals by the end of the data collection period. In case a selected file was found incomplete, it was replaced by the next file following it after randomization. We thank the reviewer for the question.

**For MTRH, the origin of the 2008-2011 files is unclear. Which “convenient sampling” was used for the 390 files of MTRH? Could the authors explain better the procedure of selection for this hospital?  How could biases be introduced for interpretability? How is representativeness kept? Please modify the methods section accordingly.**

 It was difficult to apply probability randomization as the total sampling frame could not be established. But from the total number of files that we could access from each year, the study files were chosen in a “semi-randomized” manner based on the total number of files that we could access each year and not the sampling frame. Being a non-probability sampling method, bias could be introduced. The bias will be mentioned in the limitations section. We are thankful for the comment.

**Results:**

**As mentioned by previous reviewers, the higher prevalence of KS and NHL in MTRH might reflect the tight relationship of this hospital with AMPATH/HIV. Also, the convenience sampling method used for the selection of the records for this hospital might accentuate the number of KS and NHL cases observed in this hospital.**

 We agree with the reviewer’s concern. We have highlighted this as a possible source of the study’s limitation and modified the explanation in the discussion section as suggested also by reviewer 4. We appreciate the observation.

**Please add to the current Table 7 the estimates of the 2 hospitals altogether and mention the overall 36% of cancers attributable to infectious agents.**

 We have added the estimates of the 2 hospitals to table 7. We appreciate the suggestion.

***Figures 2 & 3*** **: The titles and the figures are unclear and inconsistent with the text. From the results section, in KNH hospital, the first commonest cancers in males were prostate (11.5%) and laryngeal (9.5%) cancers. Why is colorectal cancer plotted in Figure 2? In page 9, why do the authors mention access to treatment in relation to the histograms in the result section?  Also, the different units in the y-axis are misleading. Finally, due to the small sample size by year and the use of convenience sampling for MTRH, I would suggest not to show Figures 2 and 3. Please delete the text in the results section associated with these Figures (page 9, “Proportions of the top  most cancer cases”).**

 We have deleted Figures 2 and 3 together with their associated texts in the manuscript document. We thank the reviewer for the suggestion.

***Minor comments*** **:**

**Since penile cancer is one of the cancer sites associated with infections please provide the number of cases of penile cancers that were observed in each hospital. If none were observed please provide this information in the text.**

 There were two penile cases in MTRH. The numbers have been included in table 7 in the males' section. Thank you for the suggestion.

**The authors mentioned 11 infections in page 9 but there are less infections in Table 2. Please modify accordingly.**

 We have included modified table 2 accordingly. Some infectious agents have been listed together under one cancer type. We appreciate the observation.

**Can the referral hospital for KNH be MTRH? And vice versa?**

 I strongly believe that both hospitals can receive patients referred from the other. However, I lack the evidence to support my opinion.

**Discussion:**

**As mentioned by previous reviewers, the authors gave too much emphasis on comparing the two hospital results. The presence of KS and NHL in the top four cancers for MTRH should be put in perspective with the hospital origin of the patients (HIV center). The authors should discuss this point in more detail. Given the method of selection of the patients, KNH might reflect better the overall prevalence.**

 We thank the reviewer for the comment. We have modified the infectious agents section where we have highlighted that the high numbers of KS, NHL and cervical cancer could also have been influenced by AMPATH-Oncology as the source of data for MRTH. Selection bias has been addressed in the limitation section.

**In the limitations, the representativeness of the selected patients should be addressed. How complete are the hospital registration of the patients? Could any biases be introduced at this stage? Are some cancer subtypes less likely to be recorded in these hospitals? Is misdiagnosis a potential issue?**

 We thank the reviewer for the concern. We have addressed the selection bias in the limitation section. For the 500 files that we collected, they all had the information we were aiming to get. During the data collection period, if a file had missing information, it was immediately excluded and replaced with the one following if after randomization. Convenient sampling could have led to under-representation or over-representation which we have included in the limitations section. We come across files with incomplete information or missing or even unclear diagnosis but these were excluded from the study.

*Minor comments:*

**In page 13, “Similarly an incidence using the Nairobi cancer registry...”, please correct this sentence. Do you mean incidence rates?.**

 We are thankful for the observation. We meant incidence rates and have modified the text.

**Please delete all references to the result part (Tables X, figures Y, ...) in the Discussion section.**

 Thank you for the suggestions. We have deleted the “Tables X/Figures Y” references in the discussion section.

**Summary**

**This article describes the cancer prevalence and cancers attributable to infections in two hospitals from Kenya. Due to the selection methods there are important issues to take into account when interpreting the results -** **in particular in relation to HIV-associated cancers - that have not been addressed adequately.**

 We are thankful to the reviewer for the suggestions and we have modified the text in agreement with the suggestions. We have included a text to explain or justify the high numbers of HIV- associated malignancies and a new reference. We have also highlighted the bias that could have resulted from convenient sampling or using data from AMPATH

# Peer review: Reviewer response for version 2

**DOI:** 10.21956/aasopenres.13993.r26698

**Published:** 2018-12-31

**Authors:** Maria Paula Curado

**Affiliations:** 1 Postgraduate Program in Health Sciences, Federal University of Goia´s, Goiânia, Brazil

Title

 The title is not clear with the aims ” Cancer in Kenya data from two National referral hospitals "

 It is not possible to have trends as it is a cross sectional study in two hospitals in Kenya.

Abstract

 No comments.

Introduction

 No comments.

Methods

 Why were patients aged less 18 was excluded from the study? 

 Why was clinical diagnosis not included?

 This type of study has limitations it can give more information about the hospital activities. More description about the hospital facilities (number of beds, medical, nurses, etc, radiotherapy etc ..)

Results

 Figure 1 please add the period 2008-2012 sample (500/500).

 Figure 2 this is not trends it is a proportion of cases treated in the period, it means that more patients has access to treatment in 2008 to 2011 and less in 2012. I suggest to use a histogram instead of lines for this figure and change the title.

 Why were only two cancer sites used?

 Same observations for figure 3.

Discussion

 I was expecting a comparison of your data with Eldoret and Nairobi cancer registry.

 The discussion is long for the data, please make it shorter.

 ASR are only possible to generate if you have a population based cancer registry database.

I confirm that I have read this submission and believe that I have an appropriate level of expertise to state that I do not consider it to be of an acceptable scientific standard, for reasons outlined above.

## References

[1] : The role of hospital-based cancer registries in low and middle income countries-The Nigerian Case Study. *Cancer Epidemiol*.2012;36(5) : 10.1016/j.canep.2012.05.010 430-5 10.1016/j.canep.2012.05.010 22704971PMC3438360

# Response

**Published:** 2019-02-25

**Authors:** Lucy Wanjiku Macharia

**Affiliations:** University of Nairobi (Alumni), Kenya

**Authors’ Response to Reviewers’ Comments:V2**

**Journal:** AAS Open Research

**Manuscript #:** 12910

**Manuscript Title:** Cancer in Kenya: types and infection-attributable. Data from two national referral hospitals

 We thank the reviewer for having reviewed our manuscript, for the suggestions and for the positive criticism. We have revised the manuscript in agreement with the suggestions. The discussion section has been edited to shorten the section. We hope that this new version of the manuscript can be considered for approval. Kindly find below point-by-point response to the comments and queries.

 Sincerely,

**Lucy Wanjiku  Macharia**

**Comments**

**1: Title**

***“The title is not clear with the aims” Cancer in Kenya data from two National referral hospitals"***

***It is not possible to have trends as it is a cross sectional study in two hospitals in Kenya.”***

**Response:**

 We have modified the title to make the aim clear and have removed the “trends” from the title. We are thankful for the suggestions

**2: Abstract**

***“No comments”.***

**Response:**

 We are thankful for the positive feedback

**3: Introduction**

***“No comments”.***

**Response:**

 We are grateful for the positive feedback.

**4: Methods**

**4.1 “Why were patients aged less than 18 excluded from the study.”**

**4.2 “Why was clinical diagnosis not included.”**

**4.3 “This type of study has limitations it cannot give more information about the hospital activities. More description about the hospital facilities (number of beds, medical, nurses, etc, radiotherapy etc.”**

**Responses:**

 We are thankful for the questions. There was no objective exclusion of patients under the age of 18 years. It was mainly to circumvent possible ethical debates revolving the use of data from under age group.

 All the cases had a clinical diagnosis but for them to be included in the study, a confirmatory diagnosis besides clinical, was prerequisite.

 We agree with the reviewer’s concern. An improved description of the hospital facilities has been provided in the methodology section of the new manuscript. However, to get the exact information additional permission would need to be sought from the Hospitals.

**5: Results**

**5.1. “ Figure 1 please add the period 2008-2012 sample (500/500).**

**5.2. “Figure 2 this is not trends it is a proportion of cases treated in the period, it means that more patients have access to treatment in 2008 to 2011 and less in 2012. I suggest to use a histogram instead of lines for this figure and change the title.**

**5.3. “Why were only two cancer sites used?”**

**5.4. “Same observations for figure 3”**

**Responses:**

 We are thankful for the suggestions.

 The period 2008-2012 sample (500/500) has been added in Figure 1.

 We agree with the reviewer’s concern and have modified Fig. 2 and Fig. 3 into a histogram and changed their titles.

 We used two cancer sites as they are the only ones who gave permission to access their data among all the four referral hospitals targeted at the time of the study. The reasons for refusal by the other hospitals are unknown.  

**6: Discussion**

**I was expecting a comparison of your data with Eldoret and Nairobi cancer registry. The discussion is long for the data, please make it shorter. ASR are only possible to generate if you have a population based cancer registry database.**

 We are grateful for the suggestion. The discussion section has been modified to shorten the section. There are limited published studies on “Cancer in Kenya” and the few mentioned were among the few obtainable.

 Specifically, Reference 37 used the Nairobi Cancer Registry data

                      Reference 41 used the Eldoret Cancer Registry data

                      Reference 40 The study was done at MTRH          

                      Reference 39 is a Kenya National cancer control strategy report

# Peer review: Reviewer response for version 1

**DOI:** 10.21956/aasopenres.13980.r26613

**Published:** 2018-10-01

**Authors:** Clement A Adebamowo, Michael Odutola

**Affiliations:** 1 Greenebaum Comprehensive Cancer Center, Institute of Human Virology, University of Maryland School of Medicine, Baltimore, MD, USA; 2 Institute of Human Virology Nigeria, Garki, Abuja, Nigeria; 3 Center for Bioethics and Research, Ibadan, Nigeria

**Overview**

 This paper provided information on the prevalence of cancers and the proportion of cancers that were attributable to infections in Kenyatta National Hospital (KNH) and Moi Teaching and Referral Hospital (MTRH), Kenya over a 5 year period from 2008 to 2012. These 2 centers are the oldest and largest national referral hospitals in Kenya. The authors randomly selected some cancer cases reported at these centers within the study period and reviewed the charts for cancer types and numbers of cancers attributable to infections. The study reported that 40.0% to 53.2% of cancers that were seen in these referral hospitals were attributable to infections.

**Title**

 In this paper, the numbers and proportions of cancers associated with infections were presented and not the proportion of cancers attributable to infections. In order to obtain the proportion of cancers that were attributable to infections, the author would need to apply the formula for Population Attributable Fraction (PAF) which was not done in this paper. 

**Introduction**

 The introduction is repetitive and should be edited. Separate paragraphs should be devoted to those infections that are established as causes of cancer while those will weaker associations should be in a separate paragraph.

 The role of HIV infection in cancer etiology, prevalence and incidence in Kenya deserves more discussion than just mentioning HIV in a list of pathogens. 

 Previous studies of infection-associated cancers in Kenya, East Africa or Sub-Saharan Africa should be discussed briefly to provide more introduction to the field and establish why this study was necessary and what it hopes to accomplish. 

**Methods**

 It would be more informative to describe the demographic characteristics of the catchment areas, the pattern of healthcare services including other hospitals, and referral systems for the two hospitals included in this study. While these are the biggest hospitals, they may not see the most cancer patients if specialized cancer hospitals are located in the same region or city. 

 The rationale, application, and choice of sample size calculation are unclear. This is a retrospective case series. The researchers should analyze all the case records that they can find irrespective of sample size calculation. Further, it is highly unlikely that the prevalence of cancer in adult Kenyan population is 50%.

 The justification for randomly selecting case notes to review is not clear. Rather than statistically estimate the number of files, why not obtain the number of patients seen and the number who have a cancer diagnosis directly from the institutions? If this is not possible, the authors should say so.

 The randomization procedure is not clear. The authors state that “the first record was selected randomly every year”. What is the “first record”? Record of the first patient seen in the institution within a specific year? The first record selected from among all the patients’ records for a specific year? If the latter how was the selection done? Were all the records assigned numbers and random number generators used to select records?

 Do the authors have an objective assessment of the completeness of the records of these two institutions?

 What is the relationship of the two institutions that refused to grant permission for this study and their locations with the institutions that gave permission for the study? A map showing this information would be informative for international readers

 The analytic methods used in the paper and described in the data analysis section should be described better. 

**Results**

 The authors should be precise in reporting results. “ Around 4304 inpatient cancer cases were available in MTRH” is imprecise and unacceptable.

 There is a clear difference in the cases of cancer at the KNH compared to MTRH, most probably because one site hosts AMPATH HIV treatment and prevention programs. It is therefore not justifiable to make too much of the differences in the proportion of cancers seen in these two institutions and attempt to extrapolate that to the general population.

**Discussion**

 The interpretation of the result is not justifiable based on the data, methods, and analyses. 

 Proportions of cancers presenting in specific institutions may not reflect population level epidemiology of the cancers. See Chapter 1 of the Cancer in Africa (Ed) Max Parkin et al.  

 The authors suggest that their data shows the incidence of the cancers that they described but this is not correct. 

 The authors can describe the pattern of cancers presenting to each of these hospitals over a period of time, but this is not “incidence” of those cancers in the population. The pattern of presentation of cancers to specific hospitals is influenced by many factors. An example is illustrated in their paper where the pattern of cancers presenting at the AMPATH related hospital reflects the focus on HIV treatment and prevention at the institution.

**General comments**

1. There are several grammatical and typographical errors in the paper. For example in the Discussion section, Paragraph 3 “Elsewhere, a study aiming determine the burden and pattern of cancer in …….”
2. There is inconsistency in the format of data presentation. For example, age groups were written as 22 to 44 years, and in another 45-65 years and elsewhere 45-64 years. 
3. Some sentences are too long. E.g. "Nearly 15% of the global cancer burden is attributable to infectious agents, with two-thirds of infection attributable cancers occurring in the less developed countries and in which infections accounts for nearly one in four cancers."

**Summary**

 In this paper, the authors described the prevalence of cancers and the proportion of cancers that were associated with infections in two referral hospitals in Kenya.

 The data was over-interpreted. The methods are inadequately described and the results are poorly presented. The conclusions drawn from the data are not justifiable.

We confirm that we have read this submission and believe that we have an appropriate level of expertise to state that we do not consider it to be of an acceptable scientific standard, for reasons outlined above.

# Response

**Published:** 2018-10-28

**Authors:** Lucy Wanjiku Macharia

**Affiliations:** University of Nairobi (Alumni), Kenya

**Authors’ Response to Reviewers’ Comments:**

**Journal:**          AAS Open Research

**Manuscript #:** 12910

**Manuscript Title:** Burden of cancer in Kenya: types, infection-attributable and trends. A national referral hospital retrospective survey

 We thank the reviewers for having reviewed our manuscript, for the suggestions and for the positive criticism. We have performed a major revision in the manuscript in agreement with the suggestions. We have applied the PAF/AF standard formula in estimating the number of cancer cases attributable to infections. Secondly, we have conformed to the third edition of the International Classification of Diseases for Oncology (ICD-O) way of listing the cancers. For cancers like leukemias and lymphomas where the ICD-0-3 topographical code was not clear, we converted the IDC-0-3 morphological code to ICD10 topographical code for clarity.

 We hope that this new version of the manuscript can be reconsidered to be of an acceptable scientific standard.

 Kindly find below a point-by-point response to the comments and queries.

  Sincerely,

  L.W.M

**Overall Comment:**

***“This paper provided information on the prevalence of cancers and the proportion of cancers that were attributable to infections in Kenyatta National Hospital (KNH) and Moi Teaching and Referral Hospital (MTRH), Kenya over a 5 year period from 2008 to 2012. These 2 centers are the oldest and largest national referral hospitals in Kenya. The authors*** ***randomly selected some cancer cases reported at these centers*** ***within the study period and reviewed the charts for cancer types and numbers of cancers attributable to infections. The study reported that 40.0% to 53.2% of cancers that were seen in these referral hospitals were attributable to infections.”***

**Response:**

 We are thankful to the reviewers for revising our manuscript, for the positive criticism and for the suggestions. We have revised the manuscript text, tables, figures and reference list as described below and in the revised manuscript. We have also updated our results and discussions section together with additional analysis as suggested.

**Main comments**

**1: Title**

***“In this paper,*** ***the numbers and proportions of cancers associated with infections were presented and not the proportion of cancers attributable to infections. In*** ***order to obtain the proportion of cancers that were attributable to infections, the author would need to apply the formula for the Population Attributable Fraction (PAF) which was not done in this paper.”***

**Response:**

 We agree with the reviewers’ concern. We have applied the PAF/AF standard formula in estimating the number of cancer cases attributable to infections. We are thankful for the suggestion.

**2: Introduction**

***“The introduction is repetitive and should be edited. Separate paragraphs should be devoted to those infections that are established as causes of cancer while those will weaker associations should be in a separate paragraph.***

***The role of HIV infection in cancer etiology, prevalence and incidence in Kenya deserves more discussion than just mentioning HIV in a list of pathogens.***

***Previous studies of infection-associated cancers in Kenya, East Africa or Sub-Saharan Africa should be discussed briefly to provide more introductions to the field and establish why this study was necessary and what it hopes to accomplish.”***

**Response:**

 We are grateful for the insightful suggestions. We have edited the introduction as recommended in the new manuscript.

 We have included a new paragraph on HIV prevalence and incidence in Kenya together with its associated cancers.

 Previous studies on infection-associated cancers in sub-Saharan Africa have been expounded in details in the data analysis section.

**Comment 3: Methods**

**3.1 “It would be more informative to describe the demographic characteristics of the catchment areas, the pattern of healthcare services including other hospitals, and referral systems for the two hospitals included in this study. While these are the biggest hospitals, they may not see the most cancer patients if specialized cancer hospitals are located in the same region or city.”**

****

**3.2 “The rationale, application, and choice of sample size calculation are unclear. This is a retrospective case series. The researchers should analyze all the case records that they can find irrespective of sample size calculation. Further, it is highly unlikely that the prevalence of cancer in adult Kenyan population is 50%.”**

****

**3.3 “The justification for randomly selecting case notes to review is not clear. Rather than statistically estimate the number of files, why not obtain the number of patients seen and the number who have a cancer diagnosis directly from the institutions? If this is not possible, the authors should say so.”**

****

**3.4  “The randomization procedure is not clear. The authors state that “the first record was selected randomly every year”. What is the “first record”? Record of the first patient seen in the institution within a specific year? The first record selected from among all the patients’ records for a specific year? If the latter how was the selection done? Were all the records assigned numbers and random number generators used to select records?”**

****

**3.5 “Do the authors have an objective assessment of the completeness of the records of these two  institutions?”**

****

**3.6  “What is the relationship of the two institutions that refused to grant permission for this study and their locations with the institutions that gave permission for the study? A map showing this information would be informative for international readers”**

****

**3.7 “The analytic methods used in the paper and described in the data analysis section should be described better. .”**

**Response:**

 3.1. We are thankful to the reviewers for the suggestions. We have included the demographic characteristics of the catchment areas together with the pattern of healthcare services in the study site section of the new manuscript. To clarify the reviewers’ concern, there are 12 health facilities in Kenya that offer cancer services; seven private hospitals, two mission hospitals and three public facilities (KNH, MTRH and Coast Province General Hospital). Because of the affordability of the cancer services, most patients opt for the public facilities resulting in congestion of the facilities and long waiting times of the patients. Patients with private insurance and the government-sponsored scheme, National Health Insurance Fund, are more likely to undergo treatment than those without insurance.

 3.2. We have edited the sample size calculation section and included detailed explanations with references in the new manuscript. We do agree with the reviewers’ concern of analyzing all the chart reviews. However, due to cost and time constraints, we were only able to analyze 500 files in each hospital, as this was the minimum necessary sample size needed to achieve the required power of the study. We have also come across an article showing that sampling is also applicable in retrospective chart review studies **[1]** but to avoid any misunderstanding **,** we have included this as one of the limitations of the study. Regarding the prevalence, we stated in the sample size calculation section that the prevalence of cancer in Kenya was unknown at the time of conducting the study. For a better clarification, a 50% prevalence (p) was used in calculating the sample size since it is known that when d =0.05 and a z=1.96, using a p of 0.5 (50%) yields the highest sample size required for cross-sectional studies **[2]** **.** A detailed explanation has been included in the new manuscript.

 3.3. We thank the reviewers for the positive criticism. For clarification, knowing the number of patients with cancer was not our only objective. We also aimed at knowing the of cancer types, the sex and age of the patients under study, their origin by birth, the method of cancer diagnosis used, year of diagnosis and whether the patient was referred from another health facility. At the time of the study, the hospital databases could not provide all the information needed and therefore we had to use the files. Randomization was done because of the cost and time constraints. In a facility like KNH that had 17,584 cancer files, abstracting all the information needed from the patient files to the data collection form would cost more and need longer time that the time allocated to us by the ethics committee.

 3.4. We agree with the reviewer’s concern. We have modified the randomization section.  For clarification, the files in KNH were captured in a database and randomization was purely an automated process since all files have a hospital number (that cannot be disclosed to protect the privacy of the patients). However, in MTRH, the records department was in the middle of updating their databases when this study was being done and because of this we were only given an estimate of the number of files and to achieve the required sample size we used convenient sampling method.

 3.5. Yes, the authors had an objective assessment of the completeness of the records at the two institutions. First, randomization depended on the total number given to us of the number of cancer files available for the five year period. Secondly, to obtain all the data for the questions we had, we needed to use a patient file with complete information.

 3.6. We thank the reviewers for the question. The four hospitals initially selected for the study included; Kenyatta National Hospital (KNH), Moi Teaching and Referral Hospital (MTRH), Jaramogi Oginga Odinga Teaching and Referral Hospital (JOOTRH) and Coast General Hospital previously known as Coast Province General Hospital (CPGH). At the time, they were all teaching and referral hospitals.  KNH and MTRH are national hospitals (level 6) while JOOTRH and CPGH are level 5 hospitals. KNH is located in the former Nairobi province, MTRH is located in the former Rift Valley province, JOOTRH is located in the former Nyanza province and CPGH is located in the former Coast Province. Although we had ethical approval together with a letter of authority from the Ministry of Health giving us the authorization to conduct research in the four facilities, permission to access the files was only granted by the National hospitals. We have included a map in the new manuscript to guide international readers. We are very grateful for the suggestion.

 3.7. We are thankful for the suggestion. We have edited the “data analysis” section in the new manuscript.

**Comment 4: Results**

**“The authors should be precise in reporting results. “Around 4304 inpatient cancer cases were available in MTRH” is imprecise and unacceptable.”**

**“There is a clear difference in the cases of cancer at the KNH compared to MTRH, most probably because one site hosts AMPATH HIV treatment and prevention programs. It is therefore not justifiable to make too much of the differences in the proportion of cancers seen in these two institutions and attempt to extrapolate that to the general population.”**

**Response:**

 We appreciate the suggestion. We have improved the precision of reporting the results.

 We thank the reviewers for the suggestion. We have edited the results section and minimized extrapolating the differences from the two facilities to the general population.

**Comment 5: Discussion**

***5.1. “The interpretation of the result is not justifiable based on the data, methods, and analyses.***

******

***5.2. Proportions of cancers presenting in specific institutions may not reflect population level epidemiology of the cancers. See Chapter 1 of the Cancer in Africa (Ed) Max Parkin et al.***

***5.3. The authors suggest that their data shows the incidence of the cancers that they described but this is not correct.***

***5.4. The authors can describe the pattern of cancers presenting to each of these hospitals over a period of time, but this is not “incidence” of those cancers in the population. The pattern of presentation of cancers to specific hospitals is influenced by many factors. An example is illustrated in their paper where the pattern of cancers presenting at the AMPATH related hospital reflects the focus on HIV treatment and prevention at the institution.”***

**Response:**

 5.1. We thank the reviewers for the positive criticism. We have modified the analysis and improved on the interpretation of the data. We have emphasized where necessary that we were comparing our hospital-based results to other results obtained from population-based studies.

 5.2. We agree with the reviewer’s concern that the proportions of cancers presenting in specific institutions may not be reflective at a population level. We have highlighted this as a limitation of this study.

 5.3. The use of the term “Incidence” was an unintended mistake. We have edited the discussion section and thank the reviewers for the observation.

 5.4. We agree with the reviewer’s observation. We have edited our new manuscript accordingly and avoided the use of the term incidence.

**Comment 6: General comments**

1. ***There are several grammatical and typographical errors in the paper. For example in the Discussion section, Paragraph 3 “Elsewhere, a study aiming determine the burden and pattern of cancer in …….”***
2. ***There is inconsistency in the format of data presentation. For example, age groups were written as 22 to 44 years and in another 45-65 years and elsewhere 45-64 years. ***
3. ***Some sentences are too long. E.g. "Nearly 15% of the global cancer burden is attributable to infectious agents, with two-thirds of infection attributable cancers occurring in the less developed countries and in which infections accounts for nearly one in four cancers."***

 

**Response:**

 6.1. We thank the reviewer for the observation. We have improved on the wording of the manuscript and corrected the typographical errors.

 6.2. We thank the reviewer for the comment. We have ensured consistency in the data presented in the new manuscript.

 6.3. We thank the reviewer for the suggestion. We have minimized the use of long sentences in the new manuscript.

**Comment 7: Summary**

***“In this paper, the authors described the prevalence of cancers and the proportion of cancers that were associated with infections in two referral hospitals in Kenya.***

***The data was over-interpreted. The methods are inadequately described and the results are poorly presented. The conclusions drawn from the data are not justifiable.”***

**Response:**

 We thank the reviewer for the positive criticism. We have performed major revision in the new manuscript taking all suggestions into account. We have applied the standard formula of calculating the cancers attributable to infections. We have updated the methodology section and we have ensured that the results or tables are listed according to the third edition of the International Classification of Diseases for Oncology (ICD-O). We have only reported the results as generated from the data from the two hospitals and highlighted where necessary that we were comparing our hospital-based data with other population-based studies. We have inserted a new paragraph on the limitations of this study and modified the conclusion. We are thankful for the suggestions.

**References**

 1.        Gearing RE, Mian IA, Barber J, Ickowicz A. A methodology for conducting retrospective chart review research in child and adolescent psychiatry. J Can Acad Child Adolesc Psychiatry. 2006;15: 126–134. doi:10.1186/1478-4505-4-26

 2.        Arya R, Antonisamy B, Kumar S. Sample size estimation in prevalence studies. Indian J Pediatr. 2012;79: 1482–1488. doi:10.1007/s12098-012-0763-3
